# Book Reviews

**Published:** 1914-05

**Authors:** 


### BOOK REVIEWS.

Books mentioned may be inspected at and ordered through this office. So far as possible, books received in any month will be reviewed in the issue of the second month following.

**A Reference Handbook of the Medical Sciences**; embracing the entire range of Scientific and Practical Medicine and Allied Science. By various writers. Third edition, completely revised and rewritten. Edited by Thomas Lathrop Stedman, A. M., M. D. Complete in eight imperial quarto volumes. Volume III. 934 double-column pages, illustrated by 659 engravings and 7 full-page plates in colors. $7 cloth, $8 leather, $9 half morocco. Wm. Wood & Co., New York.

The third edition of the Reference Handbook maintains the high standards of the preceding editions. This volume contains 539 articles, some very brief, as in the case of drugs of slight use, names of botanic and zoologic groups, many elaborate monographs. The brief articles are perhaps equally valuable since they virtually extend the usefulness of the Handbook to include the function of a dictionary. Biography and history are included in the scope of the work, as well as the various branches of medical science proper. The publishers have done their part of the work well. The colored plates are especially good. The two Buffalo editors of this journal are included in the list of authors. A work of this sort—

which is equivalent to saying **this work**—should be placed on a readily accessible shelf and used as a dictionary and text book for reference.

---

**Formulaire de Therapeutique Clinique,** Dr. L. Pron, with the collaboration of Dr. A. Cantonnet, in ophthalmology. Second edition, revised and enlarged, Librairie Maloine, Paris. 544 pages, 6 francs.

In this compend, the alphabetic arrangement by diseases, etc., is employed. It includes, however, such topics as altitude, marine climate, sea baths. Diseases of the eyes, ears, etc., are considered in special sections, as are poisons, a review of clinical chemistry, vaccines, etc. An alphabetic index of drugs concludes the volume. We strongly advise our readers who can read French to use this book as a convenient way to acquire hints as to practical therapeutics, in which the French excel.

---

**Communicable Diseases,** Public Health Bulletin No. 62, by Asst. Surgeon-General J. W. Kerr of the Public Health Service and A. A. Moll, A. B. Published by direction of the Surgeon-General.

This is a paper-bound volume of 700 pages, containing an analysis of the laws and regulations in force in the United States, as well as considerable matter of general interest. It is estimated that 300,000 deaths and 3,000,000 cases of com municable diseases may be obviated by proper precautions.

---

**Pathfinders of Physiology.** J. H. Dempster, A. B., M. D., Editor of the Detroit Medical Journal, published by the Detroit Medical Journal Co. 66 pages, illustrated.

This is a scholarly presentation of historic medicine, the biographies being carefully selected to mark the progress of this science. This method impresses us as much more instructive than mere antiquarian interest in medicine or than the study of the medical history of a given period. The first chapter deals with the circulation and especially with the discoveries of Harvey, Asellius and Pecquet. Digestion involves the discussion of several chemists and physiologists of the 17th and 18th centuries, and the classic investigations of Beaumont on gastric fistula, as well as those of Claude Bernard on the glycogenic function and vaso-motor nerves. In respiration and the nervous system, medical history goes back much farther, while cellular physiology, though anticipated in the 17th century, is mainly the work of the 19th. Enough biography is included in this work to enable the reader to feel acquainted with the personality of the men discussed, perhaps to understand how they reached the significant results which made them famous. But Dr. Dempster's book is not essentially a series of biographies nor a chronicle of events. He has selected men and discoveries merely as landmarks in the logical progress of physiologic science. He teaches the history of physiology by biographies, somewhat as a master of medicine or surgery might teach principles by a logical sequence of selected cases in a clinic. Thus his book is not so much a record of events as a study of forces. For a work of a historic nature, in medicine, this is almost a unique method. It gives the reader not only a grasp of the subject but more than a hint as to how medical history ought to be presented.

---

**Ethnozoology of the Tewa Indians.** Junius Henderson and John Peabody Harrington. Bulletin No. 56 of the Smithsonian Institution, Bureau of American Ethnology.

This is a study of the various forms of animal life indigenous to or introduced into the region, with the Indian names for them, whenever obtainable. Some interesting sidelights are cast upon the native language, especially in regard to the formation of names, and the indication of age, sex, etc. As in our own vernacular, the Indian sometimes confused many species or even genera under a general word, sometimes used distinct names for closely related forms of life. For many of the smaller and less conspicuous animals he had no name at all, just as we fail to recognize objects in nature which are neither especially useful, nor harmful, nor striking. Studies of this sort are of the greatest value, especially because the habits of life depend so largely upon the lower animals available for food, or other economic use, or which suggest superstitions or which are sources of danger. It is all the more important that these studies should be made soon, before aboriginal customs are too strongly modified by civilization and before the fauna of a region is too much altered by the annihilation of certain species and the introduction of others.

---

**A Synopsis of Medical Treatment.** George Cheever Shattuck, M. D., Boston, published by W. M. Leonard, Boston, 2d edition, revised and enlarged, 96 pages, $1.25.

This is a brief compend, based on the accepted practice at the Massachusetts General Hospital. Cardiac Insufficiency, Nephritis, Acute Infectious diseases, and a Synopsis of Drugs, comprise the chapters, various tables following. 23 drugs, vaccine virus, typhoid vaccine, tuberculin, normal salt solution and alcoholic beverages are singled out for special mention; 14 more drugs are merely listed as valuable for occasional use and 20 in common use. The chapters are more inclusive than mere mention might indicate; for instance, under cardiac insufficiency, most of the common forms of heart disease are considered.

There are two ways of regarding a work of this nature. It affords the student a good, practical conception of certain principles of therapeutics applicable to the common diseases which he will be called upon to treat, and a good working familiarity with a few drugs and other therapeutic measures. The same principles can be extended and the armamentarium can be increased, almost without limit. Such a work is like a geography which gives the states, 20 or 30 cities, ten or twelve rivers and the Great Lakes. The student is not bewildered either with a mass of detail or with a variety of salient points which he cannot remember. He is inspired with self-confidence and he is well prepared to get a rating of 75% on 75% of the problems with which he will ultimately be confronted.

On the other hand, the comfortable narrow horizon which shuts in his little world inevitably magnifies the relative importance which he will attach to himself, the problems which he realizes, and the means with which he is prepared to combat those problems. At the same time, the infinity of the real distances in medical science, the real weakness of the individual, the importance of the problems which are not close at hand and encountered daily, the value and the immense variety of the means with which a solution of these problems must at least be attempted, the futility of tabular views in any science, are concealed by the horizon. We do not pretend to say which of these standpoints of criticism should be adopted, but leave the decision—if a decision can be made—to those more expert in medical pedagogy.

---

**Buffalo State Hospital.** 23d Annual Report to the State Hospital Commission, for the year ending September 30, 1913.

In spite of 354 discharges, the patients increased from 2,025 to 2,063, the average daily population exclusive of paroles being 1993, the rated capacity of the hospital being 1,684. With perhaps unconscious humor it is stated that "excluding the number who died, namely 150, the destination of other patients discharged was as follows: home with members of family 162; to friends or suitable occupation 14; for deportation 20; transferred to other hospitals or homes 8." The condition of patients on discharge is better than in the Metropolitan district and practically all patients discharged are on parole, reporting regularly as to progress, needs and troubles. 18 major operations and 95 spinal punctures were performed, and about a thousand intercurrent diseases or non-operative surgical conditions independent of the mental condition were encountered. About 120 patients were confined to bed, on the average. 5 cases of typhoid, 35 of tuberculosis, and a considerable number of other acute infections were treated. A number of interesting autopsy and clinical reports are included. The yearly per capita cost was a few cents less than $200. A brief history of the institution is presented, including a biography of the late Dr. J. B. Andrews, who preceded Dr. Arthur W. Hurd as Superintendent. We congratulate Dr. Hurd—and still more so the patients and the state at large— on the economy and efficiency of the management of the hospital. Reports are usually dry and uninteresting reading. This one, without undue prolixity or the inclusion of extraneous matter, is permeated with good will and kindly interest in colleagues and patients. Without attempting to present in full the technical scientific work done at the hospital, it contains much of special professional interest.

---

**Chemical Pathology.** Being a Discussion of General Pathology from the Standpoint of the Chemical Processes Involved. By H. Gideon Wells, Ph. D., M. D., Professor of Pathology in the University of Chicago and in Rush Medical College, Chicago. Second Edition, thoroughly revised. Octavo of 616 pages. Philadelphia and London: W. B. Saunders Company, 1914. Cloth, $3.25 net.

The first edition of 549 pages has been a favorite with the writer since 1907. Most of the attention to pathology has been along the lines of histology or of perversions of physiology without definite regard to chemic conditions. The study of pathology from the strictly chemic standpoint throws light on many problems and even from the clinical standpoint, it should not be neglected. It should, indeed, be realized that the present author is dealing rather with clinical phases of the subject than with recondite analytic methods. In other words his book is a presentation of principles rather than of chemic details. Having made something of a hobby of chylous exudates, we beg leave to call attention to the fact that there are a good many interesting details that might be added to this section. The section on zootoxins presents a phase of pathology on which most medical books are silent, and which is of great interest though, fortunately academically so to most physicians of this part of the country. Few realize, for example, the terrible virulence of the sea snakes of the Indian Ocean, far exceeding that of the rattler or the cobra. The rarity with which such snakes have the opportunity to bite human beings renders them almost an unknown quantity. We understand that poisonous sea snakes of the same species exist on both sides of the Isthmus of Panama—an unaccountable fact, if it is a fact. We trust that, in future editions, the zootoxins of certain corals and other fixed animals will be considered. Being so fortunate as to possess a specimen of the duck bill, that curious mamman which lays eggs and which is so nearly extinct that it has no commercial value as a fur-bearing animal, we are interested to know that the male has poison fangs on its hind feet, and thus to be able to identify the sex of our specimen. Apropos of the statement that most snakes have a strongly toxic serum—even when not provided with poison fangs—a possibly significant value is given to the knowledge of furriers that the duck bill is not attacked by moths.

In calling attention to certain rather unusual topics, we have perhaps given the impression that Wells has failed to follow the beaten paths of pathology. Such is not the case. The various pathologic processes are systematically described but from a standpoint which has been too much neglected. While chemistry of certain secretions and excretions, of exudates, etc., has long been applied for diagnostic purposes and while quite satisfactory discussions of the chemistry of certain conditions, as gout, diabetes, etc., are commonly included in medical literature, the general study of pathology along chemic lines has not attracted the attention of the profession to the extent which it deserves. The present book, therefore, opens up a field for speculation and yields a harvest of information which should be gathered.

---

**A Manual of Clinical Diagnosis by Means of Laboratory Methods**, Charles E. Simon, B. A., M. D., Baltimore, eighth edition, 809 pages, 185 engravings, 25 plates. $5.00. Lea & Febiger, Philadelphia and New York.

The seventh edition contained 778 pages, 168 engravings and 25 plates, and was published in 1911. Simon's Clinical Diagnosis has been a standard work from the appearance of the first edition, when it appealed to the medical public solely on its merits. With added prestige, the author has relaxed nothing of his vigilance. The present edition differs from the seventh mainly in four particulars: the inclusion of the methods based on the principle of protective ferments as elucidated by Abderhalden; the revision of the technic of the Wassermann reaction; the complement fixation test in gonorrhoea; the application of modern methods of determining the existence and extent of renal disease. We bespeak for the present edition the well deserved favor which previous editions have found.

---

**Diagnostic Symptoms in Nervous Diseases.** By Edward L. Hunt, M. D., Instructor in Neurology and Assistant Chief of Clinic, College of Physicians and Surgeons, New York City. 12 mo of 229 pages, illustrated. Philadelphia and London: W. B. Saunders Company, 1914. Cloth, $1.50 net.

The first chapter deals with the examination of a nervous case. Deformities, paralysis, tremors, gaits, ataxia, convusions, reflexes, the eyes, speech disturbance, electric reactions being some of the other titles of chapters. Tabulations are frequently employed and the text is lucid and brief. This work was written at the request of many students, to obviate the trouble of hunting for diagnostic signs in more comprehensive text books. Generally speaking, the diagnosis in the sense of naming a disease technically, is of greater relative importance in neurology than in medicine generally. In a considerable number of nervous diseases, the work of the physicians is practically completed when the diagnosis is made, the condition being incurable and sometimes being best cared for in an institution. Again, the diagnosis is more apt to represent a definite pathologic condition than in general medicine, the nomenclature of neurology having sprung up coincident with pathologic advance while general medicine still uses to a large extent the earlier, empiric and popular disease names. As in other fields of practice, an accurate differential diagnosis often implies successful therapy.

---

**State Board Questions and Answers.** By R. Max Goepp, M. D., Professor of Clinical Medicine at the Philadelphia Polyclinic. Third Edition Thoroughly Revised. Octavo volume of 717 pages. Philadelphia and London: W. B. Saunders, 1913. Cloth, $4.00 net; Half Morocco, $5.00 net.

These questions are systematically grouped under chemistry, anatomy, physiology, surgery, hygiene, etc., being selected from various state board examination papers, preference being given to the larger and more representative states. The answers are brief, sometimes, therefore, somewhat incomplete and dogmatic. For example—though in each instance the answer corresponds with our own opinion—the unqualified condemnation of multiple vaccination and the omission of flies as a usual method of communicating the typhoid "poison." Reference is facilitated by an elaborate index.

---

**The Practice of Pediatrics.** By Charles Gilmore Kerley, M. D., Professor of Diseases of Children, New York Polyclinic Medical School and Hospital. Octavo of 878 pages, 139 illustrations. Philadelphia and London: W. B. Saunders Company, 1914. Cloth, $6.00 net; Half Morocco, $7.50 net.

Particularly commendable in this book is the practical and somewhat heterodox discussion of the subject of infant feeding. These quotations give an insight into the common sense views characteristic of the book: "I am confident......that of ten average girls in any station of life, provided they could have the benefits of fresh air and good food from infancy to adolescence, successful nursing mothers could be made of eight...... The most successful nursing age is 20-35. I have seen successful nursing—in a girl of 14, in a woman of 52, in the much abused society girl, while I have seen it fail in peasant women...' We were going to criticize the author for the spelling rachitis but find it supported by Webster, although it ought to be rhachitis. The labor of any author in paediatrics is increased by the tendency to include in the specialty any medical condition occurring in a child. This does not seem to be any more logical than to include fractures of limbs, pneumonia, etc., in works on gynaecology. Of course, any disease to which children are especially liable or which is substatially modified by age factors, should be included. However, the present demand is that an author in paediatrics should write a nearly complete practice of medicine and this task has been well performed in the present volume.

---

**Medical Gynecology.** By S. Wyllis Bandler, M. D., Adjunct Professor of Diseases of Women, New York Post-Graduate Medical School and Hospital. Third Thoroughly Revised Edition. Octavo of 790 pages, with 150 original illustrations. Philadelphia and London: W. B. Saunders Company, 1914. Cloth, $5.00 net; Half Morocco, $6.50 net.

This work may be considered a reaction to the gynaecology of a quarter of a century ago or more, before the term came to imply major surgery. Save for the names of the organs on which he operates, and the sex of his patients, the modern gynaecologist is, for the most part, an abdominal surgeon, and the technic which he has developed and the experience which he has acquired leads to the question whether it is not a violation of general principles of economics that he should limit his work to these particular organs and to patients of one sex. While experience taught that much of the early gynaecology was "puttering" and even dangerous, either in the sense of neglecting conditions demanding radical operation or in the direct sense, the reactionary opinion has also been growing, that many individual cases might better be treated by palliative methods, at least provisionally, and that the less radical methods of the older gynaecology may, when properly applied achieve radical cures. Our armamentarium has also been considerably improved, especially against the venereal diseases and even certain forms of sepsis which play so important a part in gynaecology. Thus, a genuine field has been reopened for conservative gynaeocology, deserving at least the respectful attention of surgical gynaecologists and, with proper precautions adapted to the general practitioner and even to specialists who do not care to enter into major surgery. This book is, therefore, timely. It is carefully written, it is conservative in its conservatism, in no way justifying the rashness of the man too cowardly to operate. We are glad to note that the author heads one chapter with the work Colpitis; sorry that he uses the word vaginitis as a legitimate synonym instead of introducing it merely to prevent any misunderstanding of the proper term; doubly sorry that further, he uses the abominable term endocervitis without apology or suggesting a correct term.

---

**The Principles of Pathologic Histology.** By Frank B. Mallory, M. D., Associate Professor of Pathology, Harvard Medical School and Pathologist to the Boston City Hospital. Octavo of 677 pages, with 497 figures containing 683 illustrations, 124 in colors. Philadelphia and London: W. B. Saunders Company, 1914. Cloth, $5.50 net.

In contrast with the book by Wells, reviewed above, this deals with structural changes as characteristic of disease. Without explicit claim, perhaps even without realization of how well he has performed the task, Mallory seems to us to have presented a work on the general philosophy of pathology. Having recently gone over one of the older standard pathologies, we are particularly impressed with the simplification of ideas as represented even in the table of contents of this book. With due apology, it may be said that pathology does not appear to be so scholarly and recondite a subject as formerly. Even the newer terms represent a more definite understanding and, especially in the chapter on tumors, a conception of mutual relations which reduces the number of individual and separate items to be remembered. The conception of inflammation stands about as it has for a quarter of a century, except that the attempt to limit it to bacterial cause is abandoned and it is better and more broadly understood. With the general simplification due to more definite knowledge, we note, paradoxically, an abandoning of the somewhat far-fetched attempts at rigorous classification. In particular, the antithesis between retrograde and proliferative processes is absent and we do not even find the orthodox discussion of hypertrophy and hyperplasia although these subjects are considered to some extent in other places. Instead of the expected section on bacteriology, we find various bacteria, protozoa, even the trichinella (nee trichina) spiralis, etc., discussed under the non-committal heading of special injurious agents.

---

**A Treatise of Diseases of the Skin.** For the use of advanced Students and Practitioners. By Henry W. Stelwagon, M. D., Professor of Dermatology, Jefferson Medical College, Philadelphia. Seventh edition, thoroughly revised. Octavo of 1250 pages, with 334 text-illustrations, and 33 full-page colored and half-tone plates. Philadelphia and London: W. B. Saunders Company, 1914. Cloth, $6.00 net; Half Morocco, $7.50 net.

This work has been so well known in its earlier editions that it is scarcely necessary to review it in detail. It will be noted that its title definitely, though courteously, separates it from the numerous medical books published at present with the aim of reaching knowledge by an easy short cut. It is a full, systematic treatise, thoroughly up to date, intended for the use of those who wish full details and who are willing to give time and serious attention to the subject. None the less, it is the kind of book needed by the general practitioner if he attempts to deal with this specialty at all, for a little knowledge is dangerous and one who relies on text books for reference needs full instruction and good pictorial aids, even more than the expert.

---

Cervical Rib. R. Waterhouse reports a case of cervical rib in which in addition to atrophy of the small muscles of the left hand, there was paresis of the serratus magnus on both sides. Prior to the operation, at which a rudimentary rib on each side was removed, there was slight irregular pyrexia which suggested the presence of inflammatory changes in the nerves of the brachial plexus as a result of pressure.—*Bristol Med. Chir. Journ.*, Sept. 1913.

